# Granular Computing Classification Algorithms Based on Distance Measures between Granules from the View of Set

**DOI:** 10.1155/2014/656790

**Published:** 2014-03-06

**Authors:** Hongbing Liu, Chunhua Liu, Chang-an Wu

**Affiliations:** School of Computer and Information Technology, Xinyang Normal University, Xinyang 464000, China

## Abstract

Granular computing classification algorithms are proposed based on distance measures between two granules from the view of set. Firstly, granules are represented as the forms of hyperdiamond, hypersphere, hypercube, and hyperbox. Secondly, the distance measure between two granules is defined from the view of set, and the union operator between two granules is formed to obtain the granule set including the granules with different granularity. Thirdly the threshold of granularity determines the union between two granules and is used to form the granular computing classification algorithms based on distance measures (DGrC). The benchmark datasets in UCI Machine Learning Repository are used to verify the performance of DGrC, and experimental results show that DGrC improved the testing accuracies.

## 1. Introduction

Granular computing (GrC) is computing method based on the partition of problem space and is widely used in pattern recognition, information system, and so forth. Zadeh identified three fundamental concepts of the human cognition process, namely, granulation, organization, and causation [1, 2]. Granulation is a process that decomposes a universe into parts. Conversely, organization is a process that integrates parts into a universe by introducing operation between two granules. Causation involves the association of causes and effects. Information granules based on sets, fuzzy sets or relations, and fuzzy relations are computed in [3]. In general, the fuzzy inclusion measure is induced by granule and union granule, such as the positive valuation functions of granules that are used to form the fuzzy inclusion measure [4–6]. But there are some problems; for example, the fuzzy inclusion measure between two atomic granules is zero no matter how far between two atomic granules is. These studies enable us to map the complexities of the world around us into simple theories.

GrC based algebraic system is a frame computing paradigm that regards the set of objects as granule, and the union operator and meet operator are the two keys of GrC. The union operator and meet operator are related to the shapes of granule. There are granules with different shapes, such as hypersphere granules, hypercube granules, hyperdiamond granules, and hyperbox granules.

The present work uses distance measure between granules with the same shapes from the view of set. A granule is represented as a vector, and the distance between granules is defined by the centers of granules and the granularities, such as the half length of hyperdiamond diagonal, the radii of hypersphere, the half length of hypercube side, and the length of hyperbox diagonal. The granular computing classification algorithms based on distance measure (DGrC) are proposed.

The rest of this paper is presented as follows. Granular computing classification algorithm based on distance measure is described in Section 2.  Section 3 demonstrates the comparative experimental results on two-class and multiclass problems.  Section 4 summarizes the contribution of our work and presents future work plans.

## 2. Granular Computing Classification Algorithm Based on Distance Measure

For the dataset *S* = {(*x*_*i*_, *y*_*i*_) | *i* = 1,2,…, *n*} in *N*-dimensional space, we construct granular computing classification algorithms (GrC) in terms of the following steps. Firstly, the single point in *S* is represented as the atomic granules which are indivisible. Secondly, the distance between two granules is proposed based on the view of set. Thirdly, the distance and granularity determine the union process jointly. Finally, the granule set is obtained and used to predict the class of unknown datum.

### 2.1. Representation of Granule and Granularity

In reality, the shapes of granules are irregular, the distance between two granules is not easily measured, the union granule, and the meet granule are related to the shapes of granules. In order to study granular computing, the granule is represented as regular shapes, such as hyperdiamond, hypersphere, hypercube, and hyperbox, especially diamond, sphere, cube, and box in 2-dimensional space. These four shape granules are represented as follows.

1. Hyperdiamond granule is represented as a vector including the hyperdiamond's center and the half of diagonal length.
2. Hypersphere granule is represented as a vector including the center and the radii of the hypersphere.
3. Hypercube granule is represented as a vector including the center and the half of side length of the hypercube.
4. Hyperbox granule is represented as a vector including vectors induced the beginning points and the end points.

Granularity is the size of granule, such as the half of diagonal length of hyperdiamond granule, the radii of hypersphere granule, the half of side of hypercube granule, and the maximal diagonal of hyperbox. The granularity of granule *G* is represented as *g*_*r*_(*G*).

For hyperdiamond granule

(1a) $$\begin{array}{c} g_{r}\left(G\right)=r, \end{array}$$

where *r* is the half of diagonal length of hyperdiamond granule.

For hypersphere granule *G*

(1b) $$\begin{array}{c} g_{r}\left(G\right)=r, \end{array}$$

where *r* is the radii of hypersphere.

For hypercube granule

(1c) $$\begin{array}{c} g_{r}\left(G\right)=r, \end{array}$$

where *r* is the half of side of hypercube.

The granularity of hyperbox granules *G* is defined as the distance between the beginning point and the end point. For hyperbox granule *G* = (**x**, **y**), the granularity is the distance

(1d) $$\begin{array}{c} g_{r}={||x-y||}_{2}. \end{array}$$

**Figure 1:**
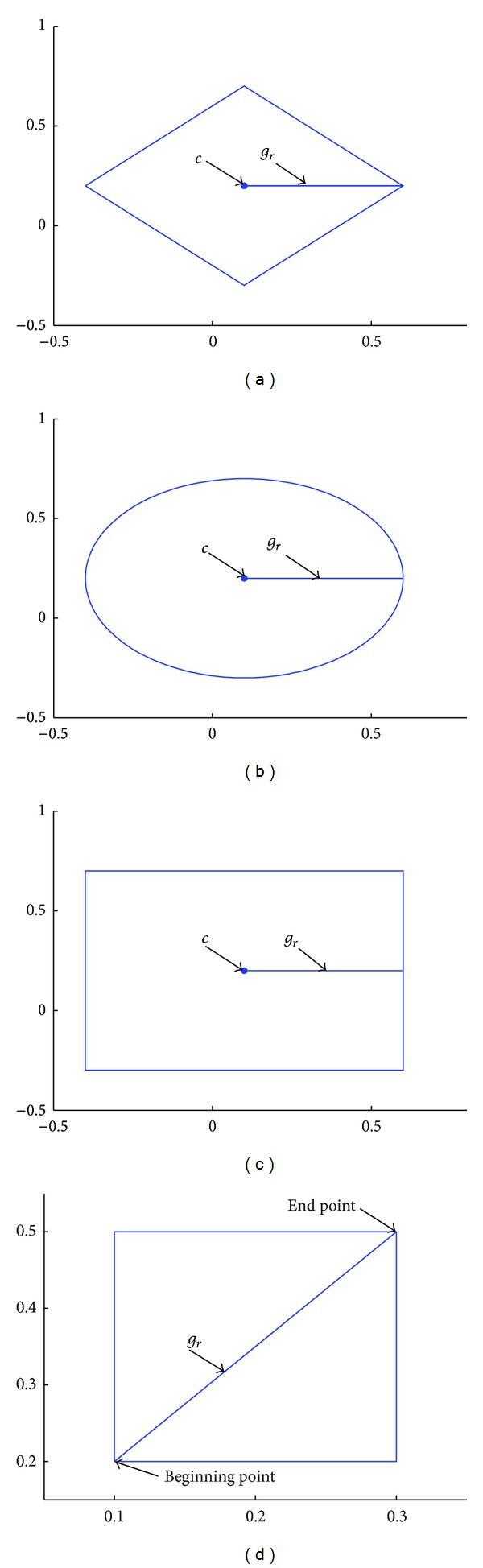
Granules with different shapes in *R* ^2^ space.

In Figure 1, *G*_1_ = (0.1,0.2,0.5) is hyperdiamond granule in *R* ^2^ space, whose center is (0.1,0.2) and granularity is 0.5. *G*_2_ = (0.1,0.2,0.5) is hypersphere granule with center (0.1,0.2) and granularity 0.5. *G*_3_ = (0.1,0.2,0.5) is hypercube granule with center (0.1,0.2) and granularity 0.5. *G*_4_ = [0.1 0.2 0.3 0.5 0.3606] is hyperbox granule with the beginning point (0.1,0.2), the end point (0.3,0.5), and the granularity 0.3606. These granules are shown in Figures 1(a), 1(b), 1(c), and 1(d). From Figure 1, we can see that different shape granules have different shapes even if they have the same forms of representations.

### 2.2. Distance Measure between Granules

The distance between granules refers to the minimal distance between two points which belong to different granules.

For two hyperdiamond granules  *G*_1_ = (*C*_1_, *r*_1_)  and  *G*_2_ = (*C*_2_, *r*_2_), the distance is

(2) $$\begin{array}{c} d\left(G_{1},G_{2}\right)={||C_{1}-C_{2}||}_{1}-r_{1}-r_{2}, \end{array}$$

where *C*_1_ = (*x*_1_, *x*_2_,…, *x*_*N*_) and *C*_2_ = (*y*_1_, *y*_2_,…, *y*_*N*_) are the centers of hyperdiamond granules *G*_1_ and *G*_2_ and *r*_1_ and *r*_2_ are granularities of hyperdiamond granules *G*_1_ and *G*_2_

(3) $$\begin{array}{c} {||C_{1}-C_{2}||}_{1}=\left|x_{1}-y_{1}\right|+\left|x_{2}-y_{2}\right|+\cdots +\left|x_{N}-y_{N}\right|. \end{array}$$

For two hypersphere granule *G*_1_ = (*C*_1_, *r*_1_) and *G*_2_ = (*C*_2_, *r*_2_), the distance is

(4) $$\begin{array}{c} d\left(G_{1},G_{2}\right)={||C_{1}-C_{2}||}_{2}-r_{1}-r_{2}, \end{array}$$

where  *C*_1_ = (*x*_1_, *x*_2_,…, *x*_*N*_)  and *C*_2_ = (*y*_1_, *y*_2_,…, *y*_*N*_) are the centers of hypersphere granules *G*_1_ and *G*_2_ and *r*_1_ and *r*_2_ are granularities of hypersphere granules *G*_1_ and *G*_2_

(5) $$\begin{array}{c} {||C_{1}-C_{2}||}_{2}=\sqrt{{\left(x_{1}-y_{1}\right)}^{2}+{\left(x_{2}-y_{2}\right)}^{2}+\cdots +{\left(x_{N}-y_{N}\right)}^{2}}. \end{array}$$

For hypercube granules  *G*_1_ = (*C*_1_, *r*_1_)  and  *G*_2_ = (*C*_2_, *r*_2_), the distance is

(6) $$\begin{array}{c} d\left(G_{1},G_{2}\right)={||C_{1}-C_{2}||}_{\infty }-r_{1}-r_{2}, \end{array}$$

where *C*_1_ = (*x*_1_, *x*_2_,…, *x*_*N*_) and *C*_2_ = (*y*_1_, *y*_2_,…, *y*_*N*_) are the centers of hypercube granules *G*_1_ and *G*_2_ and *r*_1_ and *r*_2_ are granularities of hypercube granules *G*_1_ and *G*_2_

(7) $$\begin{array}{c} {||C_{1}-C_{2}||}_{\infty }=\max\left\{\left|x_{1}-y_{1}\right|,\left|x_{2}-y_{2}\right|,\ldots ,\left|x_{N}-y_{N}\right|\right\}. \end{array}$$

For two hyperbox *G*_1_ = (**x**_1_, **y**_1_)  and  *G*_2_ = (**x**_2_, **y**_2_), the distance is

(8) d(G1,G2) =|max⁡{x11,x21}−min⁡{y11,y21}|×⋯×|max⁡{x1N,x2N}  −min⁡{y1N,y2N}|,

where **x**_1_ = (*x*_11_, *x*_12_,…, *x*_1*N*_) and **x**_2_ = (*x*_21_, *x*_22_,…, *x*_2*N*_) are the beginning points of hyperbox granules *G*_1_ and *G*_2_ and **y**_1_ = (*y*_11_, *y*_12_,…, *y*_1*N*_)  and **y**_2_ = (*y*_21_, *y*_22_,…, *y*_2*N*_) are the end points of hyperbox granules *G*_1_ and *G*_2_.

**x** = **x**_1_∨**x**_2_, **y** = **y**_1_∧**y**_2_ are operators between two vectors and defined as

(9) x1∨x2 =(max⁡⁡{x11,x21},max⁡⁡{x12,x22},…,max⁡⁡{x1N,x2N}),y1∧y2 =(min⁡⁡{y11,y21},min⁡⁡{y12,y22},…,min⁡{y1N,y2N}).

**Figure 2:**
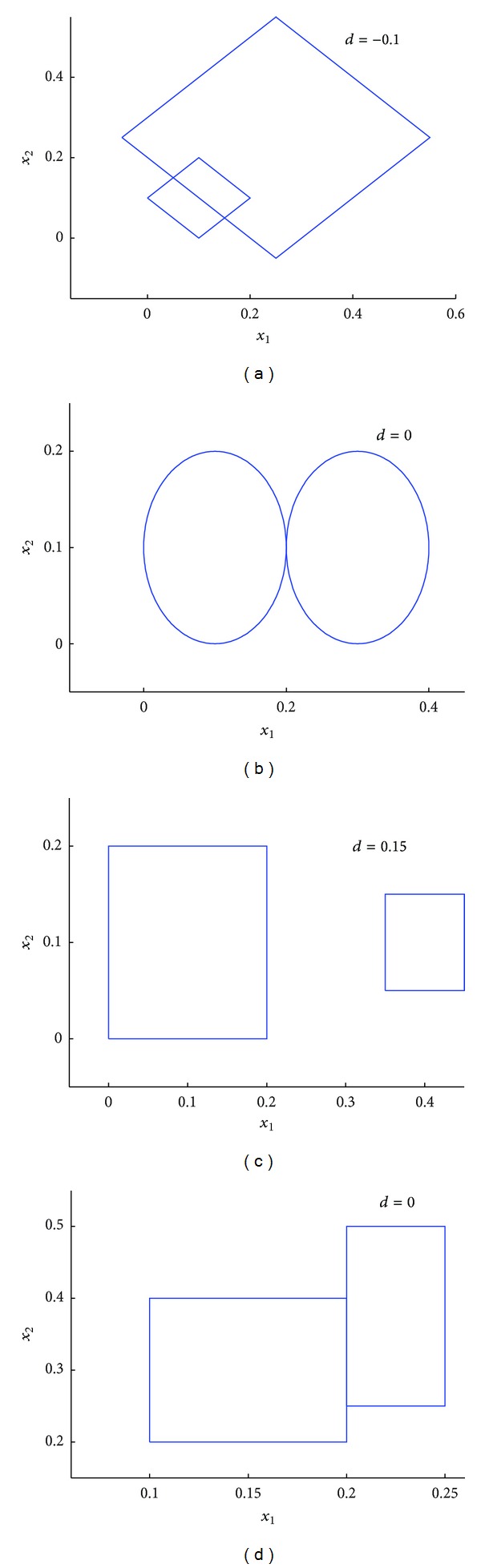
Distances between two granules in *R* ^2^ space.

According to the distance between two granules mentioned above, the distance between two granules is the arbitrary real number. There is margin between two granules when *d* > 0, there is a same point between two granules when *d* = 0, and there is an overlap between two granules when *d* < 0. When *d* > 0, the greater *d* means the greater margin between two granules, and when *d* < 0, the greater *d* means the smaller overlap.  Figure 2 shows the distance between two granules, including *d* < 0, *d* = 0, and *d* > 0.

### 2.3. Operators between Granules

Any points are regarded as atomic granules which are indivisible; the union process is the key to obtain the larger granules compared with atomic granules. Likewise, the whole space is a granule with the maximal granularity; the decomposition process is the key to divide the lager granules into smaller granules.

For two hyperdiamond granules*G*_1_ = (*C*_1_, *r*_1_)  and  *G*_2_ = (*C*_2_, *r*_2_), the union hyperdiamond granule is

(10) G=G1∨G2=(C,R),C=(12(max⁡⁡{S(:,1)}−min⁡{S(:,1)}),(max⁡⁡{S(:,2)}−min⁡⁡{S(:,2)}),…,12(max ⁡{S(:,N)}−min⁡⁡{S(:,N)})),R=12S||(id1,:)−S(id2,:)||1,

where *S* = *S*_1_ ∪ *S*_2_,  *S*_1_ = {*C*_1_ − *r*_1_ *e*_*i*_ | *i* = 1,2,…, *N*}, *S*_2_ = {*C*_2_ − *r*_2_ *e*_*i*_ | *i* = 1,2,…, *N*}, *e*_*i*_  is the vector whose *i*th component is 1, and the other components are 0,

(11) $$\begin{array}{c} id1=\arg\max S\left(:,1\right),\;\;id2=\arg\min S\left(:,1\right). \end{array}$$

For two hypersphere granules *G*_1_ = (*C*_1_, *r*_1_) and *G*_2_ = (*C*_2_, *r*_2_), the union hypersphere granule is

(12) $$\begin{array}{c} G=G_{1}\vee G_{2}=\left[C,R\right]=\left[\frac{1}{2}\left(P+Q\right),\frac{1}{2}||P-Q||\right],\;\;\; \end{array}$$

where *P* = *C*_1_ − *r*_1_(*C*_12_/||*C*_12_||),  *Q* = *C*_2_ + *r*_2_(*C*_12_/||*C*_12_||),  *C*_12_ = *C*_2_ − *C*_1_ the vector from *C*_1_ to *C*_2_.

For two hypercube granules  *G*_1_ = (*C*_1_, *r*_1_) and *G*_2_ = (*C*_2_, *r*_2_), the union hypercube granule is

(13) $$\begin{array}{c} G=G_{1}\vee G_{2}=\left(C,R\right),\\ C=\min\left\{C_{1}-r_{1}I,C_{2}-r_{2}I\right\}+R,\\ R=\max\left(C_{1}\vee C_{2}-C_{1}\wedge C_{2}\right)+r_{1}+r_{2}, \end{array}$$

where *I* is the vector with the same length as vector  *C*_1_, and all the components are 1.

For two hyperbox granules  *G*_1_ = (**x**_1_, **y**_1_) and *G*_2_ = (**x**_2_, **y**_2_), the union hyperbox granule is

(14) $$\begin{array}{c} G=G_{1}\vee G_{2}=\left(x_{1}\wedge x_{2},y_{1}\vee y_{2}\right). \end{array}$$

**Figure 3:**
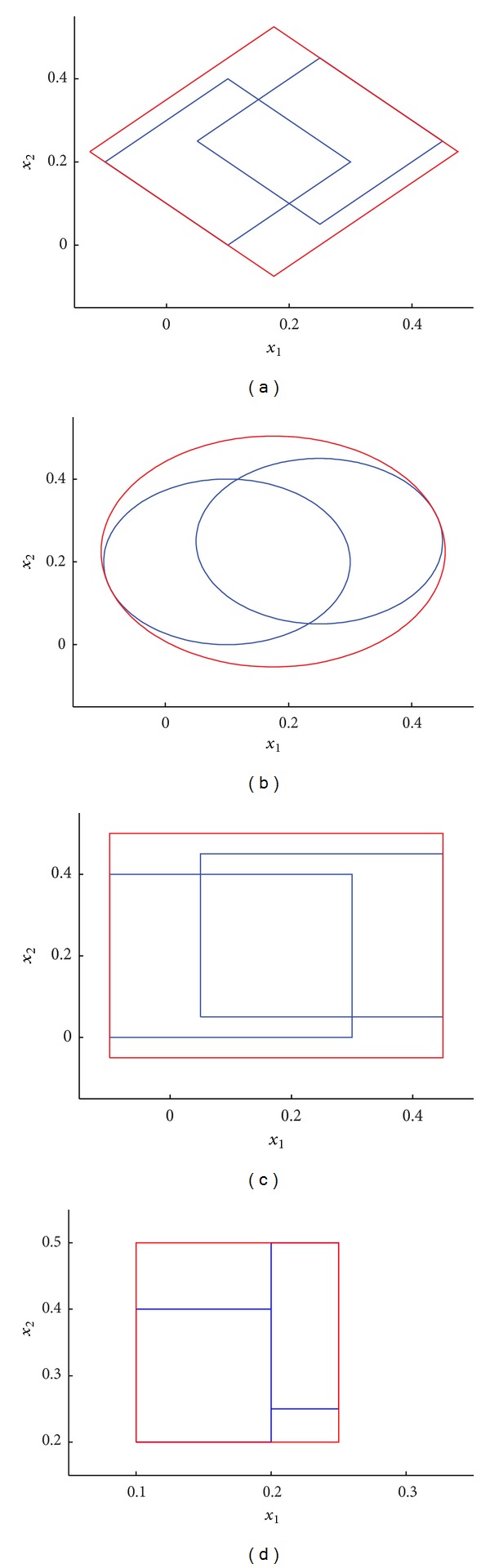
Unions between two granules. The union granules are represented as the red lines.

We explain the union process between granules in Figure 3 for 2-dimensional space *R* ^2^. Two granules *G*_1_ = [0.1 0.2 0.2] and *G*_2_ = [0.25 0.25 0.2] represent two hyperdiamond granules, hypersphere granules, or hypercube granules in 2-dimensional space, the union hyperdiamond granule is [0.1750 0.2250 0.2791], the union hypersphere granule is [0.1750 0.2250 0.38989], and the union hypercube granule is [0.1750 0.2250 0.3]. Suppose two hyperbox granules *G*_1_ = [0.1 0.2 0.2 0.4] and *G*_2_ = [0.2 0.25 0.25 0.5] in 2-dimensional space, the union hyperbox granule is [0.1 0.2 0.25 0.5]. These union granules are shown in Figure 3.

### 2.4. Granular Computing Classification Algorithms Based on Distance between Granules

The granular computing classification algorithms include two algorithms, the first algorithm is the training algorithm and the second algorithm is the testing algorithm.

For training set TS, the training granular computing classification algorithms are proposed by the following steps. Firstly, the samples are used to form the atomic granule. Secondly, the threshold *ρ* of granularity is introduced to conditionally unite the atomic granules by the aforementioned union operator, and the granule set is composed of all the union granules. Thirdly, if all atomic granules are included in the granules of GS, the union process is terminated, otherwise, the second process is continued. The training algorithm is described as follows.

**Figure 4:**
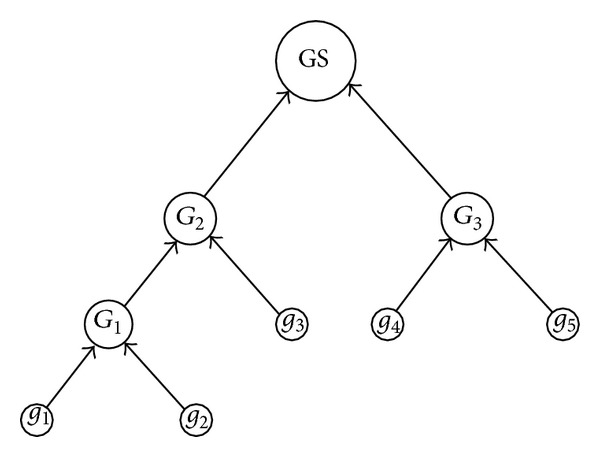
The training process of TS including 5 samples.

Suppose that the atomic granules with the same class labels induced by TS are *g*_1_, *g*_2_, *g*_3_, *g*_4_, and *g*_5_. The training algorithm can be described as the following tree structure in Figure 4; leafs denote the atomic granules, root denotes GS including its child nodes *G*_2_ and *G*_3_, *G*_1_ is induced by union operation of child nodes *g*_1_ and *g*_2_, *G*_2_ is the union granule of *G*_1_ and *g*_3_, and *G*_3_ is the union granule of *g*_4_ and *g*_5_. The whole process of obtaining GS is the bottle up process.

The threshold *ρ*, which is the cut of granularity induced by formulas (1a)–(1d) for the different shapes of granules, is selected in descending order. The larger *ρ* means the granule set induced by Algorithm 1 including the larger granules, conversely; the smaller *ρ* means the granule set induced by Algorithm 1 including the smaller granules. For the same training set, the smaller *ρ* means the induced granule set including more granules compared with the larger *ρ*.

The purpose of training algorithm is to obtain the granule set and the corresponding class lab, which are used to predict the class label of an unknown datum. The testing data including multiple data and their class labels are used to form the testing set, which is used to verify the performance of granular computing algorithms. If the prediction class labels of the testing data are same as the real class labels, the testing data are classified. Otherwise, the testing data are misclassified. The classification accuracy is one of the performances of granular classification algorithms. The testing algorithm is described as Algorithm 2.

## 3. Experiments

We evaluated the effectiveness of DGrC on both two-class and multiclass problems using Intel PIV PC with 2.8 GHz CPU and 2 GB memory, running Microsoft Windows XP Professional, and Matlab 7.0. We mainly analyze and discuss DGrCs with different shape granules from training accuracy (Tr (%)), testing accuracy (generalization ability) (Ts (%)), training time (Tr (s)), and testing time (Ts (s)).

### 3.1. Two-Class Problems

The spiral classification is a difficult problem to be classified and is used to evaluate the performance of classifiers. The training data are generated by the method proposed in [7]. The training set and the testing set in reference [8] are used to evaluate the performance of GrC.

**Figure 5:**
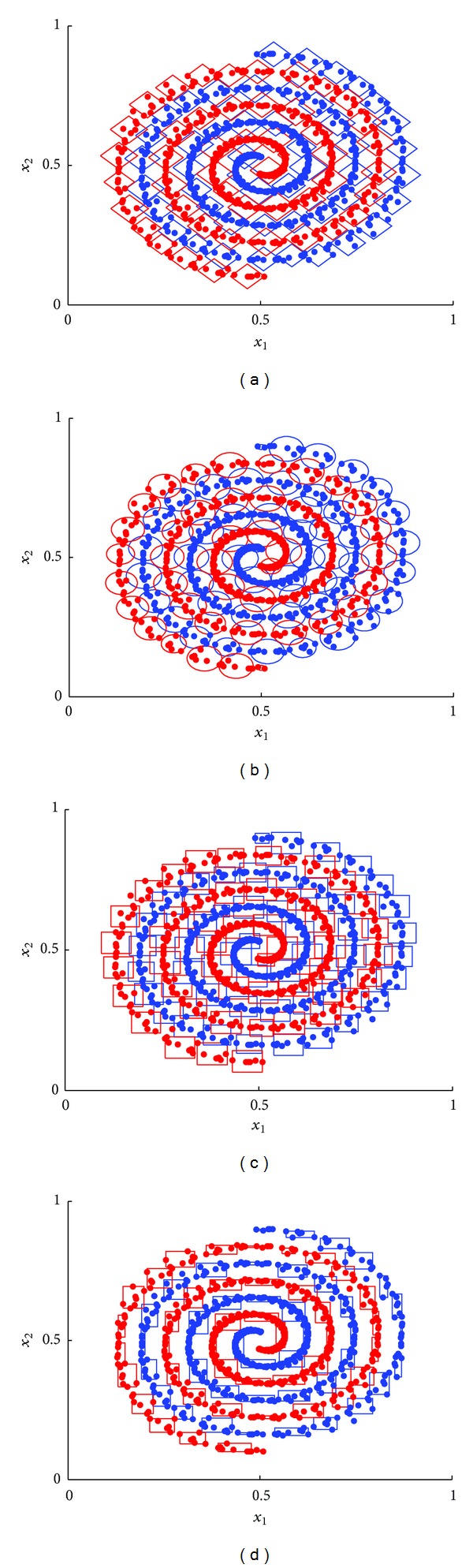
Spiral classification problem and GS (a) hypersphere granules, (b) hypercube granules, (c) hyperdiamond granules, and (d) hyperbox granules.

The threshold *ρ* of granularity is from 0.2 to 0 with step 0.001; the maximal testing accuracy is the selection indicator of optimization algorithms. Performances of GrC with four kinds of shape are listed in Table 1. The training data and their granules were shown in Figure 5 in which the single points are the atomic granules. From the table, we saw that GrC with hypersphere granules achieved the optimization performance because of the minimal size of GS including 88 granules when *ρ* = 0.094, GrC with hypercube granules is poor because of maximal size of GS including 99 granules when *ρ* = 0.079, and GrC with hyperdiamond granules touched the best testing accuracy firstly. The training time and testing time are related to the size of granule set GS, so the granular computing classification algorithms with the minimal size of granule set are our pursuits in the same conditions for the maximal test accuracy.

### 3.2. Multiclass Problems

For multiclass problems, datasets listed in Table 2 are selected from the UCI Machine Learning Repository (http://archive.ics.uci.edu/ml/) to test DGrC. They are wall-following robot navigation data (sensor2, sensor4, and sensor24) which are divided into training data and testing data at random, optical recognition of handwritten digits (optdigits) including training data and testing data, pen-based recognition of handwritten digits (pendigits) including training data and testing data, letter recognition (letter) which is divided into training data and testing data, and shuttle including training data and testing data. These datasets are used to verify the performances of DGrC from the aspects of size, Tr (%), Ts (%), Tr (s), and Ts (s) (see Table 3).

For the selected datasets, the optimal testing accuracies are 98.0769% (sensor2), 90.8691% (sensor4), 83.0220% (sensor24) 97.997% (optdigits), 97.799% (pendigits), 94.765% (letter), and 99.883% (shuttle) by KNN algorithms. We selected the optimal parameters that maximized the testing accuracy. DGrCs with 4 shapes are performed in the same environment, and the performance is listed in Table 3. From the table, we can see, for the optimal testing accuracies, that DGrC is better than KNN. (1) DGrC with hyperdiamond granules achieved the best testing accuracies 92.4092%, 87.8022%, and 99.9448%, which are highlighted by black fonts, for datasets sensor4, sensor24, and shuttle. (2) DGrC with hypersphere granules achieved the optimal testing accuracies 98.4615%, 98.1636%, 94.7953%, and 99.9448% for dataset sensor2, optdigits, letter, and shuttle. (3) DGrC with hyperbox granules achieved the optimal testing accuracy 97.9417% for pendigits.

## 4. Conclusions

The granular computing classification algorithms with different shape granules are proposed based on distance measures in the paper. Firstly, a training datum is represented as an atomic granule. Secondly, the distance measure between granules is form based on the centers and granularities of granules. Thirdly, the training process is constructed based on the union operator and the threshold of granularity jointly. Finally, the proposed granular computing classification algorithms are demonstrated by the dataset selected from references. DGrC is affected by the sequence of the training data the same as the other granular computing. For the future work, we will focus on the adaptive selection of threshold of granularities and apply the granular computing to image segmentations.

## Figures and Tables

**Algorithm 1:**
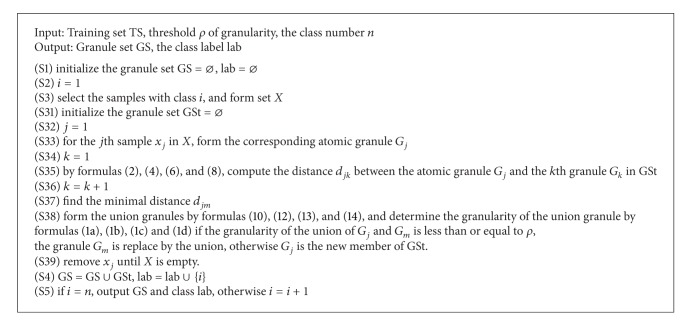
Training algorithm.

**Algorithm 2:**
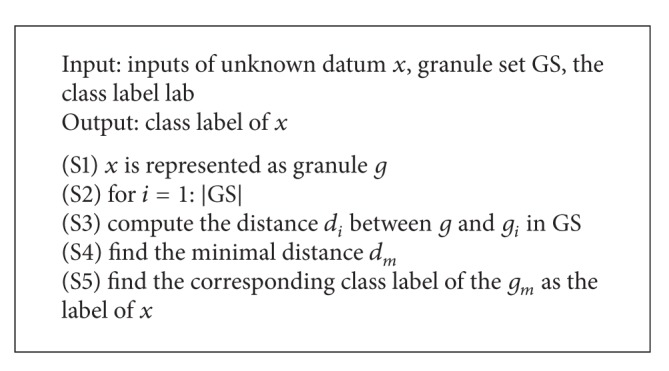
Testing algorithm.

**Table 1:** Performance of GrC with different shape granules.

Shapes	*ρ*	Size	Tr (%)	Ts (%)	Tr (s)	Ts (s)
Hyperdiamond	0.1	97	100	100	0.35938	0.015625
Hypersphere	0.094	88	100	100	0.3125	0.015625
Hypercube	0.079	99	100	100	1.4063	0.03125
Hyperbox	0.095	97	100	100	5.2656	0.0326

**Table 2:** Multiclass problems.

Data sets	Inputs	Outputs	Training size	Testing size
Sensor2	2	4	3636	1820
Sensor4	4	4	3638	1818
Sensor24	24	4	3636	1820
Optdigits	64	10	3823	1797
Pendigits	16	10	7494	3498
Letter	16	26	13333	6667
Shuttle	9	7	43500	14500

**Table 3:** Performance of DGrC on multiclass problems.

Data sets	Shapes	ρ	Size	Tr (%)	Ts (%)	Tr (s)	Ts (s)
Sensor2	Hyperdiamond	0.005	948	99.67	98.3516	1.1875	0.2031
	Hypersphere	0.0115	338	99.3674	**98.4615**	1.2031	0.1406
	Hypercube	0.01	365	99.2299	98.1319	3.8125	0.0938
	Hyperbox	0.004	1560	99.945	98.297	1.2031	0.71875

Sensor4	Hyperdiamond	0.0195	974	99.5052	**92.4092**	1.1563	0.2344
	Hypersphere	0.0085	1387	99.9175	91.7492	0.9063	0.5938
	Hypercube	0.0255	466	98.3233	90.4290	2.1563	0.1563
	Hyperbox	0.00765	906	97.306	91.474	1.5469	0.8125

Sensor24	Hyperdiamond	0.4450	2154	99.6425	**87.8022**	9.2188	2.7969
	Hypersphere	0.4450	1700	98.3773	83.2418	4.7031	2.4063
	Hypercube	0.1750	2276	99.3674	75.6593	10.0625	3.7969
	Hyperbox	0.265	2711	99.67	83.132	7.5469	6.4531

Optdigits	Hyperdiamond	1.99	3685	99.9738	97.4958	18.5625	4.813
	Hypersphere	2	1005	100	**98.1636**	2.1094	2.4063
	Hypercube	0.15	3823	100	96.3272	6.5781	12.2344
	Hyperbox	2.1	2028	99.9738	98.0523	25.0625	7.1875

Pendigits	Hyperdiamond	0.62	2334	99.97856	97.5129	5.2500	2.3281
	Hypersphere	0.28	4041	100	97.9131	5.0000	7.5000
	Hypercube	0.25	2074	99.9733	97.5129	8.8594	4.2344
	Hyperbox	0.64	5801	99.9466	**97.9417**	2.7031	9.6563

Letter	Hyperdiamond	0.13	11993	100	94.6603	7.0156	18.2188
	Hypersphere	0.065	12685	100	94.7953	3.1719	28.0469
	Hypercube	0.08	7350	100	90.2955	9.2344	19.9063
	Hyperbox	0.5	10427	98.77	94.5853	6.9844	36.8438

Shuttle	Hyperdiamond	0.0028	3052	99.9931	**99.9448**	47	10.255
	Hypersphere	0.0015	3348	100	**99.9448**	36.3125	10.8594
	Hypercube	0.00006	5895	99.9977	99.9379	58.7969	31.1250
	Hyperbox	0.0025	2920	99.9885	99.9379	26.2688	28.7813

## References

[1] Zadeh LA (1979). *Fuzzy Sets and Information Granulation. Advances in Fuzzy Set Theory and Applications*.

[2] Zadeh LA (1997). Toward a theory of fuzzy information granulation and its centrality in human reasoning and fuzzy logic. *Fuzzy Sets and Systems*.

[3] Wang L, Liu X, Pedrycz W (2013). Effective intervals determined by information granules to improve forecasting in fuzzy time series. *Expert Systems with Applications*.

[4] Kaburlasos VG, Papadakis SE (2009). A granular extension of the fuzzy-ARTMAP (FAM) neural classifier based on fuzzy lattice reasoning (FLR). *Neurocomputing*.

[5] Kaburlasos VG, Moussiades L, Vakali A (2009). Fuzzy lattice reasoning (FLR) type neural computation for weighted graph partitioning. *Neurocomputing*.

[6] Papadakis SE, Kaburlasos VG (2010). Piecewise-linear approximation of non-linear models based on probabilistically/possibilistically interpreted intervals’ numbers (INs). *Information Sciences*.

[7] Lang KJ, Witbrock DJ Learning to tell two spirals apart.

[8] Liu H, Xiong S, Fang Z (2011). FL-GrCCA: a granular computing classification algorithm based on fuzzy lattices. *Computers and Mathematics with Applications*.

